# What has changed HIV and syphilis infection among men who have sex with men (MSM) in Southwest China: a comparison of prevalence and behavioural characteristics (2013–2017)

**DOI:** 10.1186/s12889-019-7730-0

**Published:** 2019-10-21

**Authors:** Yangchang Zhang, Guohui Wu, Rongrong Lu, Wanyuan Xia, Ling Hu, Yang Xiong, Junhao Xie, Qiuhua Yu, Mengliang Ye

**Affiliations:** 1 Department of Epidemiology and Health Statistics, School of Public Health and Management, Chongqing Medical University, Chongqing, 400016 China; 2 Institute for AIDS/STD Control and Prevention, Chongqing Center for Disease Control and Prevention, Chongqing, 400042 China; 3 Department of the First Clinical Medicine, Chongqing Medical University, Chongqing, 400016 China; 4 Department of Nursing, Chongqing Medical University, Chongqing, 400016 China

**Keywords:** MSM, HIV, Syphilis, Sexually transmitted infection, Southwest

## Abstract

**Background:**

Chongqing reportedly has a large MSM population and a high STI prevalence in previous studies. However, most studies are attributed to independent cross-sectional studies, few studies have investigated trends in the prevalence of syphilis and HIV, as well as behavioural characteristics among MSM using serial surveillance surveys.

**Methods:**

Data were collected in Chongqing through face-to-face questionnaire interview and laboratory testing in Chongqing. The respondents were recruited among MSM by snowball sampling from May 2013 to December 2017. The self-report questionnaire primarily included socio-demographics, HIV knowledge, and HIV-related behaviour characteristics over the year. Blood specimens were tested to diagnose HIV and syphilis infection by Chongqing CDC. Cochran-Armitage trend test and multivariate logistic regression were conducted to compare the changes in STI prevalence and independent behavioural factors among MSM.

**Results:**

There were 6568 eligible participants (98.4%). The overall HIV prevalence was 20.5% among MSM in Chongqing, with a decrease from 23.0% in 2013 to 19.2% in 2017. The overall syphilis prevalence was 5.8%, with an increase from 3.2% in 2013 to 6.7% in 2017. The proportion of consistent condom use (CCU) during anal intercourse (46.3 to 57.7%, *P*<0.001),CCU with regular male partners(47.7 to 59.7%, *P*<0.001), CCU with casual male partners (51.5 to 62.3%, *P*<0.001) and drug use during anal intercourse (0.3 to 1.4%, *P*<0.05) were increasing. By contrast, a significant decrease was reported in the percentage of MSM with more than two regular male partners (66.0 to 21.4%, *P*<0.001) and more than two casual male partners (38.3 to 20.7%, *P*<0.001). A significant difference was observed in syphilis infection, testing for HIV antibodies and drug use during anal intercourse in the past years between the HIV-positive and HIV-negative respondents.

**Conclusion:**

A decreasing trend of HIV prevalence was showed during among MSM from 2013 to 2017 in Chongqing. While gradual reduction of high-risk behaviors along with HIV prevalence supported development of STI counselling and testing, increasing syphilis infection and drug use during anal intercourse warrants further understanding.

## Background

HIV/AIDS has arisen as the greatest concern and challenge in global public health, with a high mortality and morbidity rates [1, 2]. According to the 2017 global HIV statistics of UNAIDS, approximately 940,000 people have died from AIDS-related illnesses worldwide [3]. Despite the continuous decrease in the overall HIV incidence worldwide, an increasing trend of infection has been gradually identified among the MSM group [4]. This group was reported to have a sustained epidemic of sexually transmitted infections (STIs), such as HIV and syphilis [5]. In China, new HIV infection through homosexual transmission accounted for 12.2% of the cases in 2007, showing a dramatic increase to 25.5% in 2017 in China [6, 7]. Recent meta-analyses showed that new HIV and syphilis incidences in MSM were 5.61/100 person per year and 9.6/100 person per year, respectively, with an overall increasing trend [8, 9].

Many cohort and cross-sectional studies have reported that high-risk sexual behaviours, such as anal intercourse, inconsistent condom use, multiple partners and drug use during sex, are associated with high risk of HIV infection among MSM [10–12]. For example, high proportion of unprotected receptive anal intercourse (URAI), which is more susceptible for HIV infection than unprotected insertive anal intercourse (UIAI) was reported among MSM [10]. Notably, previous studies reported that high-risk sexual behaviour among MSM show different trends over time in China [4, 13]. The trends are partly influenced by China’s policy and economic development. For example, although marriage between males is illegal, the multicultural environment of China has increased the opportunity of this group to interact with potential sexual companions. Electronic devices have make it more convenient for young people to seek partners than the previous ways. Furthermore, the rapid economic growth and urbanization of China have accelerated the emergence of special groups, such as floating populations, immigrants and sex workers. Hence, it has become a complicated task to study the behavioural characteristics of MSM in different areas. The Chinese government has made tremendous efforts in controlling and preventing the spread of HIV. For example, the “Four Free and One Care” project was started in 2003 to support AIDS funds for non-governmental organizations (NGOs) and to conducted no threshold for antiretroviral therapy in 2016 [14, 15]. Health services (e.g., counselling, testing for antibody and free condom distribution) have also been scaled up in many cities [16]. These measures have a definite positive effect in the prevention of STIs and the intervention of high-risk behaviour among MSM.

Previously, MSM sentinel surveillance data from 37,094 people and 105 sentinel sites were conducted across 30 provinces in China. The prevalence of overall HIV infection was 6.3%, with high HIV prevalence of more than 10% reported in 24 sites across 11 provinces in 2011. The prevalence of syphilis infection was 7.8%, with 66 sentinel sites distributed over 28 provinces showing a high level of prevalence of greater than 5% [13]. A higher HIV prevalence among MSM than in other special groups have been reported in several metropolis, such as Beijing, Shanghai and Guangzhou [17–19]. However, these cities represent developed areas, data on undeveloped western areas were ignored and lacked renewal. In southwest China, both low-income and low-educational levels can be important predictors of MSM initiation and STI infection. It is important to supplement and renew data on the HIV epidemic and the changes in behavioural characteristics among MSM in southwest China.

Chongqing is the largest municipality directly under the Chinese government. It is located in southwest China, bordering Sichuan, Hubei, Hunan, Guizhou and Shanxi Provinces. Chongqing attracts a large number of migrant and floating populations from southwest China and other provinces because its bustling economy and commerce. Some studies have shown that frequent population movement can cause considerable challenges to HIV prevention and control among MSM in China [20–23]. For example, migrant workers are more inclined to seek male partners than local workers to satisfy their sexual needs, which partly results in a high infection rate of STIs in this group, and this social phenomenon is usually ignored [20]. Moreover, sex workers stay for only a few weeks in a city to avoid the ‘burned face effect’, which means that male sex workers would frequently go to another place frequently, because clients always look for new male partners at a city. These social backgrounds have increased the difficulty in AIDS surveillance [24]. According to China CDC and Chongqing CDC, new HIV infections have increased at an average annual rate of 19.7% from 2007 to 2012 in Chongqing, which was significantly beyond the national infection rate (3.13%) [25–27]. However, the data for system surveillance and epidemic trends of HIV and syphilis in Chongqing are still inconclusive..

In terms of the limited studies on MSM in Chongqing, one survey reported an average of only 34.7% participants who always used condoms with MSM, and an average of 27.5, 14.5, 29.1 and 36.2% of the MSM group lacked enough HIV knowledge from 2006 to 2009, respectively [28]. Poor knowledge and awareness can increase the risk of contracting STIs and influence the behavioural characteristics among MSM. To deeply understand the role of MSM in STIs infection and spread, this study adopted serial cross-sectional surveys to explore the prevalence of HIV and syphilis and the factors correlated with HIV among MSM from 2013 to 2017. This work also discusses the implications for intervention and assistance of this group in China.

## Materials and methods

### Study population and sample

Chongqing CDC and Chongqing Medical University formally launched HIV and STI voluntary counselling and testing (VCT) in Chongqing in 2013.With the assistance of NGOs, we initially handed out information on health services through community publicity and TV advertising. Second, snowball sampling was conducted in this investigation. We selected MSM seeds through insiders and active participants in MSM venues, such as bars, bathing pools, clubs, parks and toilets. These seeds recommended and recruited other participants to be involved in this study after completing their relevant investigation, until the recruitment process achieved adequate statistical sample size. All respondents understood the verbal declaration and signed consent forms before the study commenced.

The formula $\mathrm{n}=\frac{{{\mathrm{u}}^2}_{a/{2}^{\pi \left(1-\pi \right)}}}{\delta^2}$ was used to calculate the sample size. The HIV prevalence of HIV among MSM in 2018 in Chongqing was 21.1% [29], and the relative error (δ) was equal to 5%, α = 0.05, and u_*a*/2_ = 1.96. The *n* we calculated was 256 in an independent round. All questionnaires and laboratory examinations were conducted and distinguished by ID number and fingerprint identification. At the end of the investigation, the respondents received their test results and a gratitude fee (20 Yuan).

This investigation adopted the following inclusion and exclusion criteria. The inclusion criteria were as follows: (1) male respondents aged ≥18 years at the time of the survey; (2) male respondents who had anal intercourse with their male partners in the past year; and (3) male respondents living in Chongqing for at least a month. The exclusion criteria were as follows: (1) respondents who were not in the MSM group; (2) respondents that had mental illness or violent tendencies; and (3) respondents who failed to complete the survey questionnaire or laboratory examination. Finally, 6674 respondents were recruited in five rounds, and 6568 respondents who met the inclusion criteria were fitted into the analysis (98.4%).

### Questionnaire

The structured MSM questionnaire was administered to all participants, and face-to-face interviews were conducted in local CDC offices (see Additional file 1). To protect the participants’ privacy, trained interviewers would contact the respondent separately and ask the relevant questions according to the questionnaire outline. The interviews took an average of 30 min for each participant. The questionnaire information primarily included socio-demographic data, HIV/AIDS knowledge, behaviour of MSM, condom use, HIV testing and drug use. This questionnaire was extended as a reference to investigate the behavioural characteristics of MSM since 2008 in China [29].

In the socio-demographic section, we required respondents to fill in their age, marital status (never married/married/other), household registration (local/other city), local residence time (≤ 6 months/7–12 months/1–2 year /> 2 years), level of education (primary school or below/junior middle school/ high school / university), sexual orientation (homosexual/heterosexual/bisexual/unknown) and the place to find a male partner (physical location/Internet).

The section on gauging the participants’ knowledge of HIV consisted of nine questions, and the respondents were required to answer ‘Yes’ ‘No’ or ‘Unknown’. These questions were as follows: (1) AIDS is an incurable and serious infectious disease (2) MSM is a high-risk factor of HIV infection in China. (3) Observing the appearance can definitely judge people living with HIV.(4) STD sufferers have higher odds of HIV infection. (5) Consistent condom use (CCU) can reduce the risk of HIV transmission. (6) The usage of drugs increases the risk of HIV infection (7) HIV testing should be actively requested after the occurrence of high-risk behaviours (8) Anyone who transmits HIV intentionally would undertake legal liability. (9) You are able to assess HIV-related risks in life. According to the criteria of the structured MSM questionnaire, the respondents who answered seven or more questions correctly were regarded as having ‘eligible’ HIV-related knowledge (Cronbach’s α = 0.79).

At enrolment, the respondents were asked to report MSM-related behaviour variables. The type of sexual partner was defined as regular and/or casual, and the number was classified as a dichotomous variable.(<2 and ≥ 2). Experience of homosexual anal intercourse was collected in the past *6* months (P6M) (e.g. Yes or No). The frequency of condoms use was calculated as a trichotomous variable (e.g. never, sometimes and always) to research difference among special groups (e.g. regular/casual male partner, commercial sexual partner and female partner) (P6M). The CCU was defined as ‘Always’, and options of ‘Never’and ‘Sometimes’ were regarded as inconsistent condom use. In addition, drug use was acquired from the participants’ report (P6M) (e.g. Yes or No). Furthermore, the HIV testing variable was investigated to observe the effect of HIV/AIDS intervention. In this study, the respondents were asked whether they would assent to test for the presence of HIV antibody (P12M) (e.g. Yes or No).

### Laboratory testing

HIV and syphilis antibodies were tested among the respondents. The HIV antibody was primarily screened through enzyme-linked immunosorbent assay (ELISA). A positive result was diagnosed through Western blot assay (WB). Syphilis serum positive screening was conducted through ELISA and was confirmed through RPR and TRUST tests. All laboratory testing was completed in Chongqing CDC.

### Data quality control

The principal investigators were strictly trained to ensure that they were familiar with the questionnaire structure. Investigators assisted the respondents in answering questions based on a uniform standard. Questionnaire data were checked and logged through two people, and the data were managed using Epidata 3.0. In cases where the questionnaire data were controversial, the investigators communicated immediately with the supervisor.

### Data analysis

EpiData 3.0 software (The EpiData Association, Odense, Denmark) was used to enter and manage the data. SAS (SAS Institute, version 9.4) were utilised to describe the distribution of demographics and baseline characteristics. To quantify the annual HIV infection trends, the Cochran-Armitage trend test was employed to compare this change over the past 5 years. To identify the change in HIV-related factors, odds ratios (OR) and their 95% confidence intervals (CI) were calculated in univariate analysis. Independent variables were years from 2013 to 2017 and the dependent variables were specific behaviours (e.g. having a homosexual anal intercourse, the number of regular male partners). The OR value was the odds of exposure events with years increased. An adjusted multivariate logistic regression model was conducted to determine the adjusted odds ratio (AOR) using stepwise elimination, controlling for potential confounding and background variables. Chi-square test was utilized to examine differences in HIV-related factors between positive and negative tests over 5 years.

## Results

### Background of the study sample

**Fig. 1:**
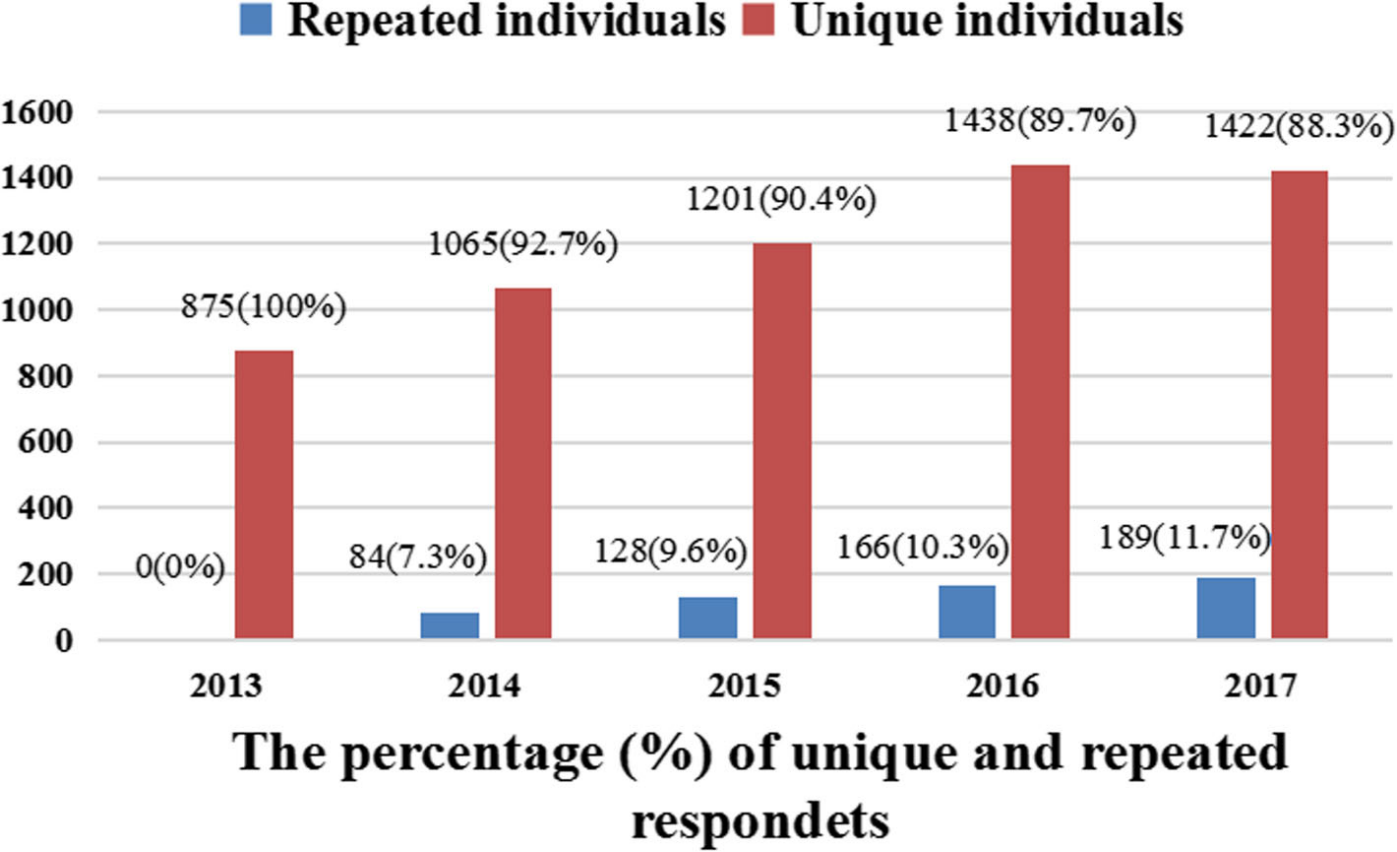
Percentage of unique and repeated respondents from 2013 to 2017

This study included 6568 adult men aged 18–77 years old. All respondents’ backgrounds were stratified by the survey rounds from 2013 to 2017. Among the socio-demographic data, 21.5% were ≤ 22 years old, 83.40% were never married, 80.2% were in Chongqing households, 98.4% were of Han nationality, 93.8% had lived in Chongqing for > 2 years, 71.9% had university degrees, 77.1% considered themselves as homosexual, 74.8% seek homsexual partners on the Internet and 85.0% achieved eligible levels of HIV-related knowledge (see Table 1). The percentage of unique individuals and repeated individuals over years are presented, respectively. (see Fig. 1).

**Table 1:** Characteristics of 6568 respondents stratified by survey year(*N*,%)

Variable	2013	2014	2015	2016	2017	Total
	*N*(%)	*N*(%)	*N*(%)	*N*(%)	*N*(%)	*N(%)*
Age (year)						
*≤ 22*	218 (24.9%)	266 (23.2%)	313 (23.6%)	313 (19.5%)	301 (18.7%)	1411 (21.5%)
*> 22*	657 (75.1%)	883 (76.8%)	1016 (76.4%)	1291 (80.5%)	1310 (81.3%)	5157 (78.5%)
Martial status						
*Never married*	716 (81.8%)	1010 (87.90)	1134 (85.3%)	1312 (81.8%)	1268 (78.7%)	5440 (82.8%)
*Married*	118 (13.5%)	86 (7.4%)	137 (10.3%)	198 (12.3%)	207 (12.8%)	745 (11.3%)
*Other*	41 (4.7%)	54 (4.7%)	58 (4.4%)	94 (5.9%)	136 (8.4%)	383 (5.8%)
Household register						
*Local*	707 (81.0%)	912 (79.4%)	1092 (82.2%)	1302 (81.2%)	1252 (77.7%)	5267 (80.2%)
*Other city*	166 (19.1%)	237 (20.6%)	237 (17.8%)	302 (18.8%)	359 (22.3%)	1301 (19.8%)
Nation						
*Han*	847 (96.8%)	1127 (98.1%)	1306 (98.3%)	1592 (99.3%)	1594 (98.9%)	6466 (98.4%)
*Other*	28 (3.2%)	22 (1.9%)	23 (1.7%)	12 (0.7%)	17 (1.1%)	102 (1.6%)
Time living in Chongqing						
*≤ 6 months*	16 (1.8%)	27 (2.3%)	24 (1.8%)	26 (1.6%)	41 (2.5%)	134 (2.0%)
*7–12 months*	14 (1.6%)	14 (1.2%)	16 (1.2%)	14 (0.9%)	14 (0.9%)	72 (1.1%)
*1–2 year*	44 (5.0%)	48 (4.2%)	41 (3.1%)	27 (1.7%)	40 (2.5%)	200 (3.0%)
*>2 years*	801 (91.5%)	1060 (92.3%)	1248 (93.9%)	1537 (95.8%)	1516 (94.1%)	6156 (93.8%)
Level of education						
*Primary or below*	12 (1.4%)	13 (1.1%)	10 (0.8%)	16 (1.0%)	12 (0.7%)	63 (1.0%)
*Junior school*	60 (6.9%)	50 (4.4%)	72 (5.4%)	111 (6.9%)	111 (6.9%)	404 (6.2%)
*High school*	175 (20.0%)	234 (20.4%)	270 (20.3%)	367 (22.9%)	333 (20.7%)	1379 (21.0%)
*University*	628 (71.8%)	852 (74.2%)	977 (73.5%)	1110 (69.2%)	1155 (71.7%)	4722 (71.9%)
Sexual orientation						
*Homosexual*	595 (68.0%)	873 (76.0%)	956 (71.9%)	1337 (83.4%)	1300 (80.7%)	5061 (77.1%)
*Bisexual*	0 (0.00%)	0 (0.00%)	0 (0.00%)	1 (0.1%)	2 (0.1%)	3 (0.1%)
*Heterosexual*	265 (30.3%)	259 (22.5%)	305 (22.9%)	245 (15.3%)	267 (16.6%)	1341 (20.4%)
*Unknown*	15 (1.7%)	17 (1.5%)	68 (5.1%)	21 (1.3%)	42 (2.6%)	163 (2.5%)
Method of seeking same-sex partner						
*Physical place*	875 (100%)	178 (15.5%)	101 (7.6%)	190 (11.8%)	309 (19.2%)	1650 (25.2%)
*Internet*	0 (0.00%)	971 (84.5%)	1228 (92.4%)	1414 (88.2%)	1302 (80.8%)	4915 (74.8%)
HIV-related knowledge^3^						
*≥ 7*	772 (88.2%)	1121 (97.6%)	1268 (95.4%)	1055 (65.8%)	1367 (84.9%)	5583 (85.0%)
*< 7*	103 (11.8%)	28 (2.4%)	61 (4.60%)	549 (34.2%)	244 (15.1%)	985 (15.0%)

Note.^1^Five-year average HIV prevalence; ^2^ Five-year average HIV prevalence.^3^ Number of correct responses to knowledge items.

### Trends of HIV and syphilis prevalence

**Fig. 2:**
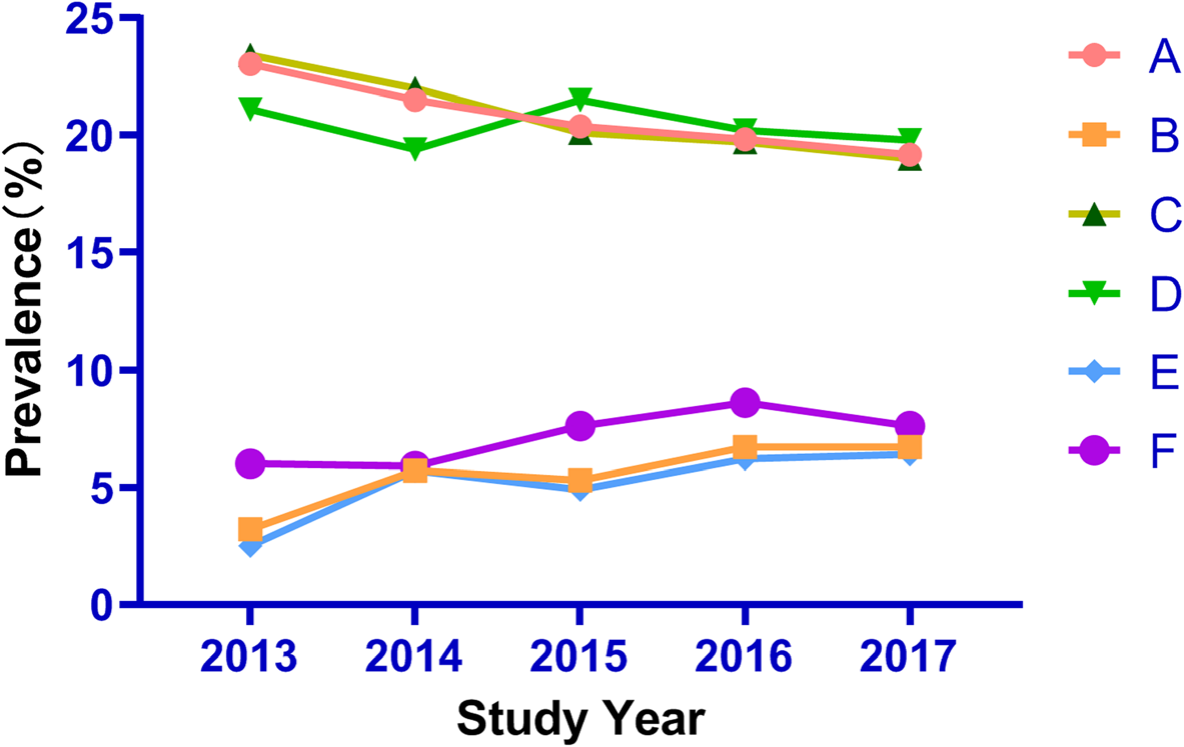
Percent of HIV and syphilis prevalence by study round and household. Red circle (A): Overall HIV prevalence;Orange square (B): Overall Syphilis prevalence; dark green triangle (C): HIV prevalence in locals; emerald green (D): HIV prevalence in non-locals; sky blue diamond: Syphilis prevalence in locals; purple circle: syphilis prevalence in non-locals

A total of 1345 respondents were HIV positive (20.5%), and 374 respondents were syphilis positive (5.8%) based on laboratory testing. Significant linear trends of HIV and syphilis prevalence among MSM were found on a 5-year basis (*p* < 0.05 and *p* < 0.01). A total of 23.0, 21.5, 20.4, 19.8 and 19.2% of the respondents were confirmed to be infected with HIV over the 5-year study period, and the Cochran–Armitage trend test was significant (Z = 2.42, *p*<0.05). A total of 3.2, 5.7, 5.3, 6.7 and 6.7% of respondents were confirmed to have syphilis infections over the 5-year study period, and the Cochran–Armitage trend test was also significant (Z = −.3.44, *p*<0.001). Generally, HIV prevalence showed a downward trend from 2013 to 2017, whereas the syphilis prevalence fluctuated at 5–6%. The average prevalence of HIV was 20.5 and 20.9% in the local and non-local groups, respectively, whereas the average prevalence of syphilis was 5.4 and 7.3% in both subgroups. The line chart is presented in Fig. 2.

### The change in behavioural characteristics among MSM

Approximately 89.3% of locals and 87.8% of the floating population admitted that they had homosexual anal sex in the previous **6** months. This proportion changed little, and a significant trend was not found from 2013 to 2017 (*P* for trend: 0.64 and 0.10). Approximately 31.9% of the locals and 30.7% of the floating population had more than two regular male partners, and this proportion decreased among the locals (65.9% in 2013 to 21.0% in 2017), as well as in the floating population (66.4% in 2013 to 22.9% in 2017). (AOR = 0.54, 95% CI: 0.51–0.56 among the locals; AOR = 0.56, 95% CI = 0.51–0.62 in the floating population). Similarly, the number of casual male partners significantly declined among the locals (38.4% in 2013 to 20.0% in 2017) and the floating population (37.8% in 2013 to 23.2% in 2017) (AOR = 0.79, 95%CI: 0.71–0.85 among the locals; AOR = 0.77, 95%CI: 0.75–0.83 in the floating population).

The proportion of CCU during anal intercourse increased among the locals (47.7% in 2013 to 57.2% in 2017) and in the floating populations (39.9% in 2013 to 59.0% in 2017) (AOR = 1.12, 95% CI = 1.08–1.17 among the locals; AOR = 1.30, 95% CI = 1.19–1.42 in the floating population). In addition, approximately 55.5% of the participants admitted to CCU with regular partners, ranging from 47.7% (in 2013) to 59.7% (in 2017), and this proportion significantly increased in both groups (AOR = 1.15, 95%CI = 1.10–1.20 among the locals; AOR = 1.26, 95%CI = 1.15–1.38 in the floating population). For CCU with casual partners, this proportion also significantly increased from 2013 to 2017 (AOR = 1.07, 95% CI = 1.01–1.14 among the locals;AOR = 1.31, 95% CI = 1.16–1.46 in the floating population). However, the proportion of CCU during commercial sexual intercourse was significant only in the locals, with a continuous increase (AOR = 0.59, 95%CI = 0.40–0.87). Few respondents had experiences with drugs, and this change was found among the locals (AOR =1.28, 95% CI = 1.05–1.54)(See Table 2**).**

### HIV-related behaviour, knowledge, and service

Homosexual anal intercourse was positively associated with HIV infection in 2013, 2014 and 2015 (*P*<0.001). Having multiple regular male partners was positively associated with HIV infection in 2017 (*P*<0.05). Meanwhile, having multiple casual male partners was potential risk factors for HIV infection in 2016 (*P*<0.05) and 2017 (*P*<0.001). CCU during anal intercourse, CCU with casual partners and CCU with regular partners were negatively associated with HIV infection from 2013 to 2017 (*P*<0.001), and CCU with females was negatively associated with HIV infection in 2015, 2016 and 2017. Syphilis infection was higher in the HIV-positive group than in the HIV-negative group during the 5-year study period (*P*<0.001). Drug use during anal sex was connected with the HIV-positive group from 2014 to 2017 (*P*<0.001). In addition, testing for the HIV antibody in the past year was considered a significant protective factor from 2013 to 2017 (*P*<0.001), and people having eligible HIV knowledge had lower HIV infection over the 5-year study period except in 2014 (*P*<0.001)(See Table 3).

**Table 2:**
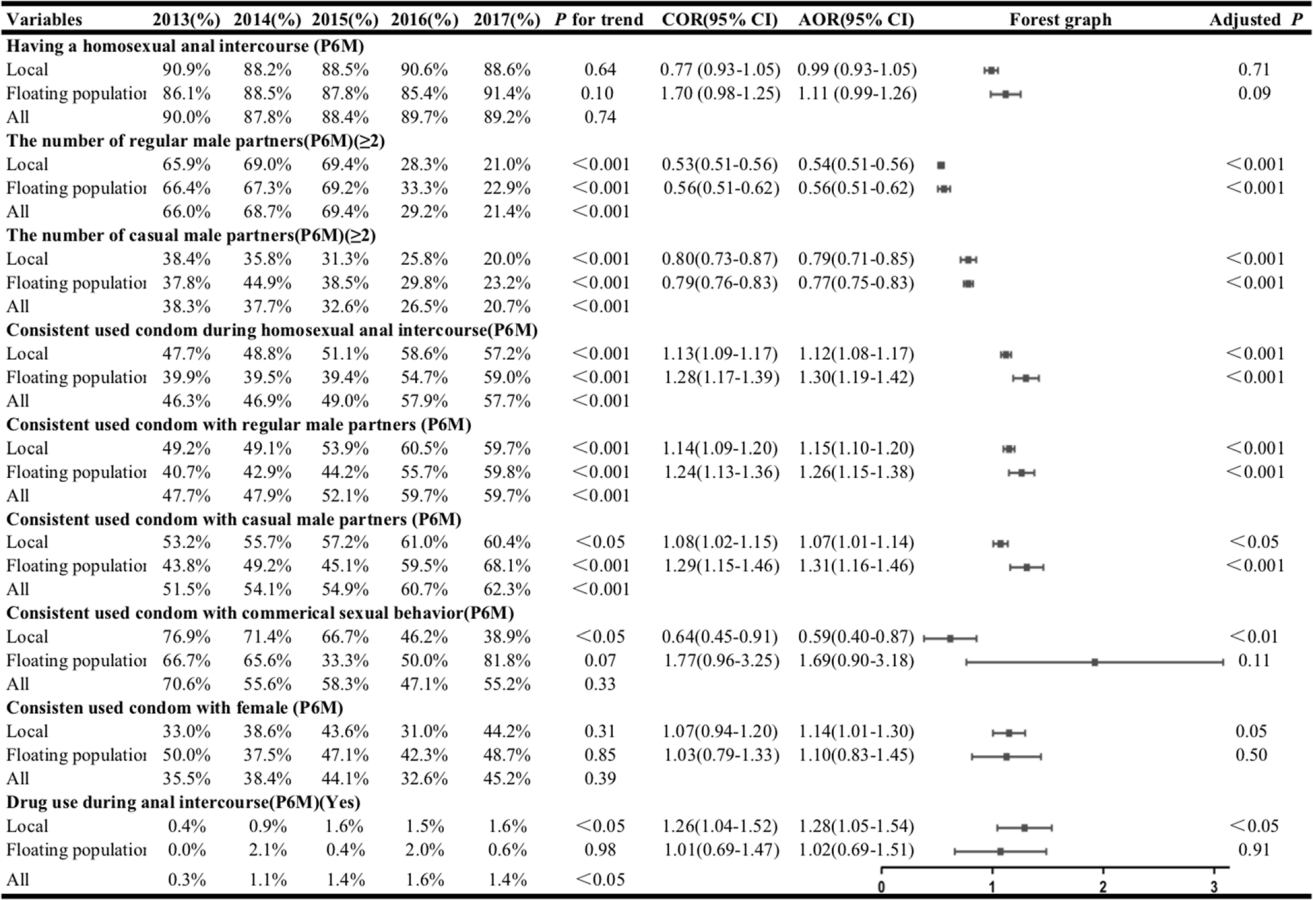
The sexual behavioural characteristics of MSM in Chongqing, 2013–2017.

**Table 3:** The change in HIV-related behaviour, knowledge and service from 2013 to 2017 in MSM, grouped by HIV status

Variable	2013	2014	2015	2016	2017
Having homosexual anal intercourse (P6M)					
Positive^1^	95.5%^***^(192/201)	93.1%^***^(230/247)	94.8%^***^(256/270)	90.60%(288/318)	88.70%(274/309)
Negative^2^	88.40%(596/674)	86.40%(779/902)	86.70% (919/1059)	89.40%(1150/1286)	89.30%(1163/1302)
The number of regular male partners (P6M) (≥2)					
Positive	63.5%(122/192)	65.2%(150/230)	65.6%(168/256)	31.9%(92/288)	26.3%*(72/274)
Negative	66.8%(398/596)	69.7%(543/779)	70.4%(647/919)	28.5%(328/1150)	20.3%(236/1163)
The number of casual male partners (P6M) (≥2)					
Positive	36.6%(218/596)	39.6%(91/230)	34.8%(89/256)	31.30%*(90/288)	29.9%***(82/274)
Negative	43.8%(84/192)	37.1%(289/779)	32.0%(294/919)	25.30%(291/1150)	18.60%(216/1163)
Consistent used condom during homosexual anal intercourse (P6M)					
Positive	24.0%***(319/596)	31.1%***(72/230)	23.8%***(61/256)	28.5%***(82/288)	28.1%***(77/274)
Negative	53.5%(46/192)	51.5%(401/779)	56.1%(515/919)	65.2%(750/1150)	64.7%(752/1163)
Consistent used condom with regular male partners (P6M)					
Positive	26.6%***(45/169)	33.9%***(64/189)	30.3%***(69/228)	32.3%***80/248()	30.3%***(70/231)
Negative	54.5%(282/518)	51.7%(356/688)	58.2%(484/832)	66.2%(689/1041)	66.3%(685/1033)
Consistent used condom with casual male partners (P6M)					
Positive	26.1%***(29/111)	30.6%***(38/124)	31.7%***(44/139)	31.9%***(46/144)	34.3%***(49/143)
Negative	61.1%(180/295)	61.5%(243/395)	61.9%(283/457)	69.0%(345/500)	69.9%(416/668)
Consistent used condom during commercial sexual behaviour (P6M)					
Positive	66.7%(8/12)	41.7%(5/12)	50.0%(2/4)	25.0%(1/4)	25.0%(1/4)
Negative	80.0%(4/5)	83.3%(5/6)	62.5%(5/8)	53.8%(7/13)	60.0%(15/25)
Consistent used condom with female partner(P6M)					
Positive	28.0%(7/25)	42.1%(8/19)	18.8%*(3/16)	17.1%**(7/41)	27.3%**(9/33)
Negative	37.6%(23/85)	37.3%(25/67)	48.4%(46/95)	37.1%(53/143)	49.0%(75/153)
Drug use during anal intercourse (P6M) (Yes)					
Positive	0.0%(0/201)	3.2%***(8/247)	4.4%***(12/270)	3.5%***(11/318)	4.9%***(12/309)
Negative	0.4%(3/674)	0.6%(5/902)	0.6%(6/1059)	1.1%(14/1286)	0.5%(7/1302)
Syphilis infection					
Positive	8.5%***(17/201)	13.0%***(32/247)	12.2%***(33/270)	16.0%***(51/318)	21.8%***(63/289)
Negative	1.6%(11/674)	3.8%(34/902)	3.6%(38/1059)	4.4%(56/1286)	3.2%(39/1238)
Testing for HIV-antibody(P12M)					
Positive	58.3%***(112/192)	62.6%***(144/230)	66.0%***(169/256)	67.7%***(195/288)	56.6%***(155/274)
Negative	71.5%(426/596)	74.5%(580/779)	81.8%(752/919)	82.3%(946/1150)	79.6%(926/1163)
Eligible HIV knowledge					
Positive	82.1%***(165/201)	96.4%(238/247)	90.7%***(245/270)	57.9%***(184/318)	77.7%***(240/309)
Negative	90.2%(607/674)	97.9%(883/902)	96.6%(1023/1059)	67.7%(871/1286)	86.6%(1127/1302)

Note. ^1^HIV-positive, ^2.^HIV-negative, ^*^p<0.05, **p<0.01, ^***^p<0.001.

## Discussion

The MSM group is a high-risk population that is of great concern in HIV/AIDS pandemic research. In this study, we examined the prevalence trends of HIV and syphilis from 2013 to 2017 among MSM in Chongqing, and changes in sexual behaviour characteristics were assessed between the local and floating population groups. Finally, we compared the compositions of HIV-related factors between the HIV-positive and HIV-negative groups over the 5-year study period. The results extend the reliability and the validity of this study by utilising a 5-year cross-sectional design. Our results showed that HIV decreased and had a high prevalence from 2013 to 2017 among MSM, but the prevalence of syphilis was lower and it increased. In the subgroup analysis, the composition of multiple sexual partners constantly decreased, and the proportion of CCU showed a growing trend. .

The overall HIV prevalence decreased from 23.0% in 2013 to 19.2% in 2017, but this rate was higher than that in the general population (0.09%) and general MSM group in China (8%) [6, 15]. The prevalence of HIV among MSM in Chongqing is significantly higher than that reported in many previous works in China. For example, previous studies were conducted in several metropolis in China. These studies reported an average infection rate of 9.5% in Harbin, 9.1% in Chengdu, 7.8% in Shanghai, 7.8% in Beijing and 14.5% in Guangzhou [17–19, 30, 31]. Moreover, nine consecutive cross-sectional surveys among MSM were conducted from 2008–-2016 in Beijing, with increasing pooled HIV prevalence ranging from 5.0 to 10.2% [32]. Internationally, the overall HIV prevalence in cisgender (non-transgender) MSM was 14.0% in eight African countries recruiting 4586 participants [33]. Although the HIV prevalence among MSM is high in Chongqing, a significant declining trend was detected over 5-year study period, with a decreasing prevalence of 10% among MSM since 2013. Notably, the higher prevalence reported in the current study may be connected with the sampling strategy of convenient sampling. Previous large studies also adopted snowball sampling to investigate behavioural characteristics among MSM [19, 30, 31]. However, some shortcoming are inevitably encountered in such research. For example, snowball sampling is a strategy of one-way incentive, which decreases the efficiency of recruitment [34]. In contrast, respondent driven sampling (RDS) is a strategy of two-way incentive, which enrolls participants from long recruitment ‘chains’, decreasing the proportion of rejection in the process of enrollment and revealing more authentic population characteristics in HIV most-at-risk population [35]. In summary, extended prevention, proactive policies and an effective sampling strategy are important in curbing the HIV epidemic. Syphilis prevalence among MSM showed a moderately rising level from 2013 (3.1%) to 2017 (6.7%). Paradoxically, a previous literature study enrolling 171,311 MSM reported that overall syphilis and HCV prevalence showed a decreasing trend (syphilis: from 9.1% in 2009 to 6.3% in 2013) across 30 provinces in China [36].

In the present work, the percentage of participants with two or more regular/casual partners showed a dramatic decline from 2013 to 2017, and multiple sexual partners were associated with a higher HIV-infection risk. Similar results were found in previous studies, suggesting that having multiple partners among MSM results in higher odds of STD infection and low CCU than the general population [30, 37–39]. Several reasons may be proposed to explain this finding. Regular male partners are considered intimate relationships that are distinguished from commercial sex workers. In this relationship, most MSM examine the image of loyalty and trust more than self-protection. Hence, it is far more difficult to persuade partners to use condoms during intercourse, and the consistent use of condoms may be regarded as mistrust of the lover [24]. In western China, approximately 62% of MSM respondents reported having anal sex with three or more partners, and the proportion of CCU was low for regular (15.8%) and casual (16.3%) partners, respectively [40]. Moreover, smartphones and the Internet provide convenient ways for young people to seek casual partners (e.g. ZANK, Jack’d and Blue); Blued is a large platform of MSM communication with > 30 million registered users [41]. In addition, male partners may play a ‘bridge’ or ‘amplifier’ role in STI transmission among MSM [42, 43]. In light of the high overall HIV prevalence in Chongqing, multiple sexual partners can increase the odds of HIV infection among MSM.As the new generation (‘Millennials’) is intent on individual expression and is not interested in listening to stereotyped propaganda and traditional ideas, there is a very urgent need for the government to innovate prevention and management means for these groups.

Interestingly, our result are inconsistent with those of previous studies reported the trend of multiple sexual partners among MSM in China. For example, the 2005–2011 surveillance results showed that the percentage of MSM who had multiple homosexual sexual partners over 6 months increased from 68.0% in 2008 to 85.4% in 2011 [13, 44]. In addition, MSM was not only associated with high-risk behaviours but also with high migration rates, which can increase the number of casual sexual partners [45]. In the present study, multiple sexual partners were negatively associated with years for the floating populations along with downward HIV prevalence. This result suggests that floating populations can be key groups to look into in further research. The Chongqing CDC has been supported by The Global Fund AIDS Project and Bill Gates AIDS Project since 2006, which has improved the HIV surveillance network, strengthened STI testing and behavioural intervention, and promoted social support for high-risk groups such as prostitutes, MSM, drug addicts and floating population.

Corresponding to previous results, the proportion of CCU in this study increased trend with regular, casual partners and anal intercourse behaviour. In a previous research, CCU with partners had a lower proportion among MSM; however, sex workers among MSM accounted for nearly 50% with CCU [7]. In the present study, we also found a relatively lower percentage of condom use with regular (overall: 47.7% in 2013 to 59.7% in 2017) and casual (overall: 51.5% in 2013 to 62.3% in 2017) partners, but this phenomenon was better than that in the past (*P* for trend: <0.001). In addition, this proportion was lower than that in reports in Guangzhou and Shanghai, and higher than that in Beijing and Harbin [19, 31, 32, 46]. Anal intercourse is radical intercourse, which increases the risk of rectal rupture and HIV/STI infection. A study conducted in three central Asian countries, namely, Kyragyzstan, Uzbekistan, and Tajikistan, and revealed that low-income status, debt, homelessness and limited access to medical care can be potential risk factors for URAI among external migrants [47]. These background factors indicate that floating populations may have great odds of high-risk sexual acts, such as receptive anal intercourse without condom use. Although MSMs are inclined to use condoms when they are at self-perceived risk [36], there were few significant changes in consistent used condom with sex workers and females in the MSM group. One possible explanation for this insignificant association is that homosexuals were the overall majority and could have weaken heterosexual effects. The second potential explanation is that seeking a male partner has become easy through physical places and the Internet, it has decrease commercial sex behaviour incidences. The third possible explanation is that the homosexual culture is prevailing among the younger group, and the social reception of this behaviour has gradually improved.

Considering the aforementioned issue, social media intervention has been used to promote social support and strengthen community connection among MSM [48, 49]. A longitudinal study suggests that social media interventions can enhance HIV services among MSM in China, with participating online local community contests to promote HIV testing [50]. In 2010, Chongqing CDC and relevant NGOs committed to construct network platforms by utilising WeChat and websites to spread knowledge and skills about HIV/AIDS prevention. Previous studies also indicated that MSMs who had URAI were willing to accept consultation and antibody testing because they had a high-risk perception [51].

Drug use only significantly increased among MSM during the study period. Drug use through injection is a major mode of STI transmission in Eastern Europe and Central Asia. In China, this problem was found in Yunnan Province where the first Chinese HIV sub-type was confirmed. A previous study reported that Jiulongpo and Yuzhong districts had high illicit drug use, with 5.8 and 9.4% in Chongqing, respectively, and the highest HIV prevalence was found in these districts, with 9.4 and 15.1%, respectively [52]. However, the drug issue is more sensitive, involving related law and ethics, and requires multi-sectoral cooperation in the future.

This study is subject to the same limitations as the others. First, the current work was a cross-sectional study and did not illustrate the causal relationship. Second, the questionnaire was answered on the basis of subjective reports and recruited respondents by snowball sampling, which may result in information bias and selective biases. Third, because MSM and HIV/AIDS are sensitive issues, some relative items were easily missed, and some respondents dropped out during the study. Fourth, only one province (Chongqing) was selected to examine the status of MSM, ignoring other southwestern Chinese provinces, such as Sichuan, Guizhou and Yunnan.

## Conclusions

The overall HIV prevalence among MSM steadily decreased over the past 5 years in Chongqing, while the pooled syphilis prevalence among MSM slightly increased during the same study period. These finding may be important predictors of HIV prevention that the proportion of participants with multiple male partners decreased and the percentage of consistent condom use increased. Moreover, eligible knowledge and testing for HIV antibody in the past year are both protective factors among MSM for HIV prevention. Syphilis infection and drug consumption are drivers of high HIV/STI prevalence. Understanding the change in the behavioural characteristics over years is a potential target for effective interventions aimed at combating the high HIV burden among MSM in China.

## Supplementary information

**Additional file 1.** The questionnaire for men sex with men: This structured questionnaire includes socio-demographic data and characteristics of sexual behaviors among MSM.

## Data Availability

The data that support the findings of this study are available from Chongqing CDC but restrictions apply to the availability of these data, which were used under license for the current study, and so are not publicly available. Data are however available from the authors upon reasonable request and with permission of Chongqing CDC.

## Abbreviations

CCU: Consistent condom use
ELISA: Enzyme linked immunosorbent assay
HIV: Human immunodeficiency virus
MSM: Men who have sex with men
RDS: Respondent driven sampling
STIs: Sexually transmitted infections
UIAI: Unprotected insertive anal intercourse
URAI: Unprotected receptive anal intercourse
VCT: Voluntary counselling and testing
WB: Western blot
