# Supplementary material for: What has changed HIV and syphilis infection among men who have sex with men (MSM) in Southwest China: a comparison of prevalence and behavioural characteristics (2013–2017)
Source: BMC Public Health. 2019 Oct 21;19:1314. doi: 10.1186/s12889-019-7730-0 (PMC6805434; doi:10.1186/s12889-019-7730-0)
Supplement: Supplementary file 1 — Additional file 1. The questionnaire for men sex with men: This structured questionnaire includes socio-demographic data and characteristics of sexual behaviors among MSM. [file 12889_2019_7730_MOESM1_ESM.pdf]

The Questionnaire for Men Who Have Sex With Men

Hello! We come from Chongqing CDC or Chongqing Medical University. We are conducting a survey to understand people's knowledge about health and the characteristic of sexual behaviors. Please be assured that this survey is anonymous and we will keep your answer confidential. We hope that your answer is your personal truth. The survey will take up about 10 minutes. At the end of the survey, I can give you some help (for example, you can ask some health questions, We will try to answer). We hope you will support our work. Thank you!

A01 Survey area:___________

A02 Survey population: MSM

A03 Zip code of survey area:___________

A03a The wave of survey:____________

A04 Questionnaire number:____________

A05 Date of survey:___________

A06 Source of sample:__________

A07 Fingerprint number:___________

B01 Birth year:__________

B02 Marital status: 1. unmarried 2. married 3.cohabiting 4. divorced or windowed

B03 Household registration 1.local 2.other city

B04 Nation:___________

B05 Local residence time: 1.(≤ 6 months) 2.(7 – 12 months) 3.(1 – 2 year) 4.(>2 years)

B06 Education: 1. primary school or below 2.junior middle school 3.high school 4.university

B07 Sexual orientation:1.homosexual 2.heterosexual 3.bisexual 4.unknown

B08 The place to find a male partner: 1.physical location 2.Internet

B09 Which software you use to seek sexual partners:_________

C01 AIDS is an incurable and serious infectious disease.

1. Yes 2.No 3.Unknown

C02 MSM is a high-risk factor of HIV infection in China.

1. Yes 2.No 3.Unknown

C03 Observing the appearance can definitely judge people living with HIV.

1. Yes 2.No 3.Unknown

C04STD sufferers have higher odds of HIV infection.

1. Yes 2.No 3.Unknown

C05 Consistent condom use (CCU) can reduce the risk of HIV transmission.

1. Yes 2.No 3.Unknown

C06 The usage of drugs increases the risk of HIV infection.

1. Yes 2.No 3.Unknown

C07HIV testing should be actively requested after the occurrence of high-risk behaviours.

1. Yes 2.No 3.Unknown

C08 Anyone who transmits HIV intentionally would undertake legal liability.

1. Yes 2.No 3.Unknown

C09 You are able to assess HIV-related risks in life.

1. Yes 2.No 3.Unknown

D00a How old were you when you first had sex with a man?_________

D00b What was your sexual role in anal sex?

1. receptive 2. insertive 3. both

D01 Have you had anal sex with your partner in the last six months?

1. Yes 2. No

D02 How many times have you had anal sex with your peers in the last week? ________

D03 Did you use condoms the last time you had anal sex with a homosexual partner in the last six months?

1.Yes 2.No

D04 How often do you use condoms during anal sex with your homosexual partner in the last six months?

1. Always 2. Sometimes 3. Never

D05 How many regular sexual partners have you had in the last six months?_______

D06 Did you use condoms when you had anal sex with regular partners in last time in the last six months?

1.Yes 2.No

D07 How often do you use condoms the last time having anal sex with regular partners in the last six months?

1. Always 2. Sometimes 3. Never

D08 How many casual sexual partners have you had in the last six months? ________

D09 Did you use condoms the last time you had anal sex with casual partners in the last six months?

1.Yes 2.No

D10 How often did you use condoms during anal sex with casual partners in the last six months?

1. Always 2. Sometimes 3. Never

E01 Have you had commercial sex with your peers in the last six months?

1. Yes 2.No

E02 How often did you use condoms when having commercial sex with your partner in the last six months?

1. Always 2. Sometimes 3. Never

E03 Did you use condoms last time you had commercial sex with peers in the last six months?

1.Yes 2.No

F01 Have you had sex with female in the last six months?

1.Yes 2.No

F02 How often did you use condoms when having sex with females in the last six months?

1. Always 2. Sometimes 3. Never

F03 Did you use condoms the last time you had sex with females in the last six months?

1.Yes 2.No

G01a Have you taken drugs in the last six months?

1. Yes 2.No

H01 Have you ever been diagnosed with venereal diseases in the last year?

1. Yes 2.No

H02 Have you received syphilis treatment in the last year?

1. Yes 2.No

J01 Have you ever been tested for HIV/AIDS in the last year?

1. Yes 2.No

J02 Do you know the test results?

1. Yes 2.No

This is the end of the investigation. Thank you for your cooperation!
